# Genome-Wide Identification of NAC Transcription Factor Family in *Juglans mandshurica* and Their Expression Analysis during the Fruit Development and Ripening

**DOI:** 10.3390/ijms222212414

**Published:** 2021-11-17

**Authors:** Xiang Li, Kewei Cai, Xiaona Pei, Yan Li, Yanbo Hu, Fanjuan Meng, Xingshun Song, Mulualem Tigabu, Changjun Ding, Xiyang Zhao

**Affiliations:** 1 State Key Laboratory of Tree Genetics and Breeding, School of Forestry, Northeast Forestry University, Harbin 150040, China; lx2016bjfu@163.com (X.L.); ckwnefu@163.com (K.C.); ly2019nefu@163.com (Y.L.); huyb200@126.com (Y.H.); mfj19751@163.com (F.M.); sfandi@163.com (X.S.); 2 College of Forestry and Grassland, Jilin Agricultural University, Changchun 130118, China; xiaonapei2020@163.com; 3 Southern Swedish Forest Research Centre, Swedish University of Agricultural Sciences, 230 53 Alnarp, Sweden; mulualem.tigabu@slu.se; 4 Key Laboratory of Tree Breeding and Cultivation of State Forestry Administration, State Key Laboratory of Tree Genetics and Breeding, Research Institute of Forestry, Chinese Academy of Forestry, Beijing 100091, China

**Keywords:** *Juglans mandshurica*, NAC family, genome-wide analysis, walnut exocarp, embryos, expression pattern

## Abstract

The NAC (NAM, ATAF and CUC) gene family plays a crucial role in the transcriptional regulation of various biological processes and has been identified and characterized in multiple plant species. However, genome-wide identification of this gene family has not been implemented in *Juglans mandshurica*, and specific functions of these genes in the development of fruits remain unknown. In this study, we performed genome-wide identification and functional analysis of the NAC gene family during fruit development and identified a total of 114 *JmNAC* genes in the *J. mandshurica* genome. Chromosomal location analysis revealed that *JmNAC* genes were unevenly distributed in 16 chromosomes; the highest numbers were found in chromosomes 2 and 4. Furthermore, according to the homologues of *JmNAC* genes in *Arabidopsis thaliana*, a phylogenetic tree was constructed, and the results demonstrated 114 *JmNAC* genes, which were divided into eight subgroups. Four *JmNAC* gene pairs were identified as the result of tandem duplicates. Tissue-specific analysis of *JmNAC* genes during different developmental stages revealed that 39 and 25 *JmNAC* genes exhibited upregulation during the mature stage in walnut exocarp and embryos, indicating that they may serve key functions in fruit development. Furthermore, 12 upregulated *JmNAC* genes were common in fruit ripening stage in walnut exocarp and embryos, which demonstrated that these genes were positively correlated with fruit development in *J. mandshurica*. This study provides new insights into the regulatory functions of *JmNAC* genes during fruit development in *J. mandshurica*, thereby improving the understanding of characteristics and evolution of the *JmNAC* gene family.

## 1. Introduction

Transcription factors (TFs) are proteins that specifically bind to cis-acting elements in the promoter regions and play a vital regulatory role in abiotic and biotic stress responses in many plants [1,2]. They can bind to specific nucleotide sequences upstream to regulate the transcription or gene expression of functional genes, which can significantly enhance stress resistance or tolerance to various factors and regulate plant growth and development [3,4]. The NAC protein family is one of the largest plant-specific TF families [5]. The family name is derived from the common proteins of no apical meristem (NAM) from *Petunia hybrida*, ATAF1–2 and cup-shaped cotyledon (CUC) from *Arabidopsis thaliana*, which prompted the formation of a conserved NAC domain [6,7]. Furthermore, the N-terminal of encoded NAC proteins possesses a highly conserved DNA-binding domain with approximately 150 amino acids and consists of five typical subdomains that are divided into A–E, wherein the A, C, and D subdomains are relatively conserved [8,9]. The C-terminal contains highly varying transcriptional regulatory regions and is specifically involved in DNA binding [10,11].

Owing to their crucial function in the formation of the embryos and flowers of petunias, NAC TFs were widely studied, and multiple reports identified its key role in plant growth and development [12,13]. A large number of NAC-encoding genes have been identified and confirmed in various plants for their role in regulating wood formation [14], leaf senescence [15], fruit development [16,17], and flower morphogenesis [18,19]. Furthermore, NAC TFs also are involved in various biotic and abiotic stress responses in many plants such as *Populus tomentosa* [20], *Arabidopsis thaliana* [21], *Oryza sativa* [22,23], *Glycine max* [24], *Arachis hypogaea* [25], and *Xanthoceras sorbifolia* [26]. The overexpression of many NAC genes increases stress tolerance in plants. In a study on *A. thaliana*, the overexpression of an NAC gene from *Malus baccata* indicated that *MbNAC25* significantly increased cold and salinity tolerance [27]. The overexpression of *ChNAC1* in *Cerasus humilis* exhibited high drought stress tolerance since it positively regulated the expression of abscisic acid (ABA)-responsive genes [27,28]. Furthermore, in *Nicotiana tabacum*, the overexpression of *LpNAC13* from *Lilium pumilum* caused significant negative regulation to drought stress tolerance but positively regulated the salt stress response [29]. The NAC family plays a crucial role in secondary cell wall biosynthesis. The overexpression of *BpNAC012* in *Betula platyphylla* positively regulated ectopic secondary cell wall deposition [30]. In *O. sativa*, *OsSND2*, an NAC TF, was involved in regulating secondary cell wall biosynthesis [31]. NAC TFs can not only regulate the expression of downstream genes but also cooperate with other TFs to regulate the target genes. Previous studies have demonstrated that *MaNAC5* cooperates with *MaWRKY1* and *MaWRKY2* for regulating pathogenesis-related gene response to diseases [32]. In addition, a study on *Glycine max* indicated that *GmWRKY27* interacted with *GmMYB174* and suppressed *GmWRKY29*, which significantly enhances drought stress tolerance [33].

Fruits are mainly involved in reproduction and gene transmission of plants, and they also provide many essential nutrients when consumed [34]. Fruit development and ripening are complex processes regulated by multiple factors such as gene regulation, hormone induction, temperature, and light. In particular, it was regulated by many transcription factors, which affected the expression of downstream target genes at the transcriptional level [35]. Previous studies have confirmed that NAC transcription factors participate in fruit development during ethylene biosynthesis and signal transduction [36]. Furthermore, some genes involved in plant hormone biosynthesis and signal transduction also are directly regulated by NAC transcription factors during fruit development and ripening process [37]. A recent study suggested that NAC TFs serve specific functions in fruit development and ripening in model species and crops of commercial interest and are involved in several cellular processes such as pigment accumulation, tissue softening, and organic acid biosynthesis [38]. In *Picea abies*, the overexpression of *PaNAC03* influences flavanol biosynthesis and embryo development [39]. Furthermore, the seed and fruit development of *Vitis vinifera* is influenced by the interaction of *VvNAC26* and *VvMADS9* [40]. Owing to their specific expression in biological processes, NAC genes are important for conferring specificity to plant growth, development and adaptability.

To date, the NAC gene family has been widely identified and studied in many species, such as *Musa acuminata* [41], *Medicago sativa* [42], *Panax ginseng* [43], *Dimocarpus longan* [44], and *Actinidia chinensis* [45]. However, no comprehensive study has been conducted on the NAC gene family of *J. mandshurica*. The recent completion of *J. mandshurica* genome sequencing (https://ngdc.cncb.ac.cn/gwh/Assembly/22547/show) (accessed on 12 October 2021) allowed us to investigate the NAC gene family of *J. mandshurica* at the genome level. In the present study, we systematically performed genome-wide identification and analysis of the NAC gene family, which bridges the research gap for gene family studies in *J. mandshurica*. The analyses of physical and chemical characteristics, chromosomal location, phylogeny and evolutionary relationships, conserved motifs, regulatory network, and expression profiling of *JmNAC* genes were comprehensively performed. This study will improve the understanding of the functional characteristics of *JmNAC* genes to identify their role in the development of walnut exocarp and embryos in *J. mandshurica* and will also lay a foundation for further study of molecular mechanisms of fruits.

## 2. Results

### 2.1. Identification and Chromosomal Location of JmNAC Genes

To identify the NAC gene family members of *J. mandshurica*, sequence alignment of NAC genes in *A. thaliana* and *J. mandshurica* was performed using two BLASTP (Basic Local Alignment Search Tool for Protein) searches. After redundancy removal, a total of 114 highly confident NAC genes were screened and identified in the *J. mandshurica* genome. Furthermore, the names of *JmNAC* genes were encoded from *JmNAC1* to *JmNAC114* according to the gene annotation information (Table S1). The basic information of these genes was analyzed and summarized in detail, including their *M*_W_, pI, CDS length, and subcellular localization. Of the identified 114 *JmNAC* genes, the largest protein was encoded by *JmNAC046* with 1160 amino acids and CDS length of 3483 bp, whereas the smallest protein was encoded by *JmNAC089* with 77 amino acids and CDS length of 234 bp. The calculated theoretical *M*_W_ of the proteins ranged from 8773.86 kDa (*JmNAC089*) to 132,712.8 kDa (*JmNAC046*), and pI varied from 4.21 (*JmNAC089*) to 9.83 (*JmNAC081*). With regard to subcellular localization, all *JmNAC* proteins were predicted, and the results showed that they may located in the nucleus (Table S1).

**Figure 1:**
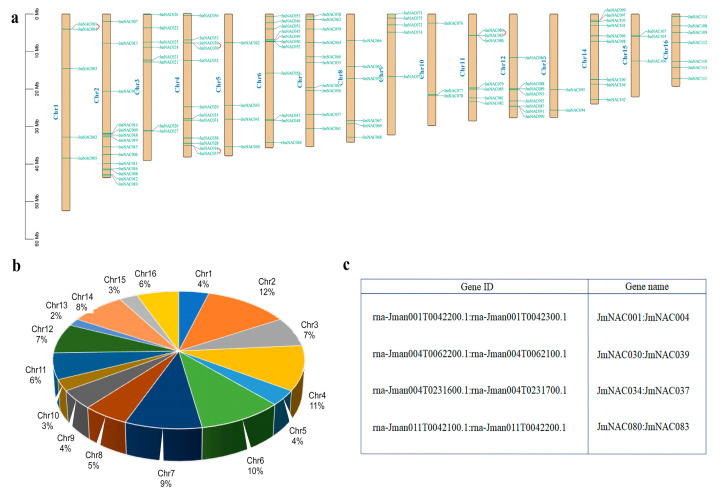
Chromosomal distribution of *JmNAC* genes on eight *J. mandshurica* chromosomes. (**a**) Distribution of *JmNAC* genes in 16 chromosomes. Tandemly duplicated genes are marked with red. (**b**) The pie chart represents the distribution of *JmNAC* genes on each chromosome. (**c**) Tandemly duplicated *JmNAC* gene pairs in *J. mandshurica*.

The identified 114 *JmNAC* proteins were unevenly distributed on 16 chromosomes of *J. mandshurica* (Figure 1a), indicating the diversification and complexity of the NAC family. Chromosome 2 contained the largest number of *JmNAC* genes (14 genes, ~12%), followed by chromosome 4 (12 genes, ~11%), and chromosome 13 contained the smallest number of *JmNAC* genes (2 genes, ~2%) (Figure 1b). In addition, a total of 5 (~4%), 8 (~7%), 4 (~4%), 11 (~10%), 10 (~9%), 6 (~5%), 5 (~4%), 3 (~3%), 7 (~6%), 8 (~7%), 9 (~8%), 3 (~3%) and 7 (~6%) genes were located on chromosomes 1, 3, 5, 6, 7, 8, 9, 10, 11, 12, 14, 15, and 16, respectively. Furthermore, *JmNAC* genes on chromosomes 2, 7, 10, 13, and 14 were mainly located at one end of the chromosome. Duplication events are of great importance for studying plant evolutionary patterns. According to the chromosomal location and genome annotation information, a total of four tandemly duplicated gene pairs were obtained, which were mainly distributed in chromosomes 1 and 4, with two gene pairs each (Figure 1c).

### 2.2. Phylogenetic Analysis of JmNAC Genes

**Figure 2:**
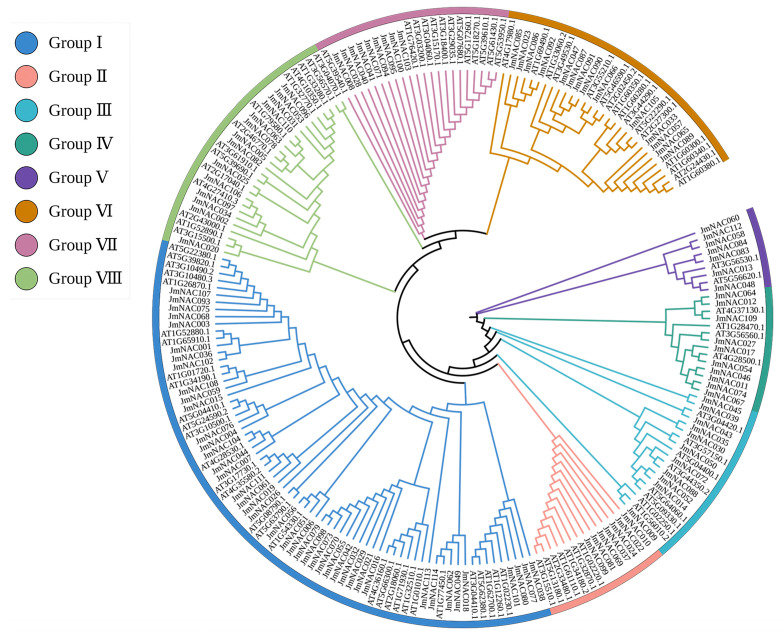
Phylogenetic tree representing the relationship between NAC genes of *J. mandshurica* and *A. thaliana*. Different colors represent the subgroups of the NAC gene family in *J. mandshurica* and *A. thaliana*.

To understand the phylogenetic relationship and potential molecular function of the *JmNAC* gene family, phylogenetic analysis was performed using all NAC full-length protein sequences from *J. mandshurica* (114 genes) and *A. thaliana* (96 genes). Based on the homology of NAC proteins, 114 *JmNAC* genes in the constructed phylogenetic tree were classified into eight subgroups, namely, Group I, Group II, Group III, Group IV, Group V, Group VI, Group VII and Group VIII (Figure 2). Group I consisted of the largest number of *JmNAC* genes, that is, 42 genes, followed by Group VI and VIII with 14 genes each. Group III and IV included 11 and 10 *JmNAC* genes, respectively, whereas Group II, VI and VII contained less than 10 *JmNAC* genes. Furthermore, *JmNAC060*, *JmNAC112*, *JmNAC058*, *JmNAC084*, *JmNAC083*, *JmNAC013*, and *JmNAC048* exhibited relatively low similarity with genes in other subgroups; therefore, they were included in Group V, representing a single clade.

### 2.3. Gene Structure, Motif Composition and Protein Interaction Analysis of JmNAC Genes

Structural differences in genes are the basis of gene family evolution, which contribute to understanding the diversity of genes and environmental adaptability in plants. To investigate the diversity of gene structure, the structure of *JmNAC* genes, including untranslated regions (UTR), exons, and introns was identified using TBtools. Genes grouped in the same class had a similar gene structure. We found that the number of exons in all *JmNAC* genes ranged from 3 to 13, and the number of introns varied from 1 to 12, exhibiting significant variation. *JmNAC046* contained the largest number of exons and introns, followed by *JmNAC084*, which consisted of 12 exons and 11 introns. Some *JmNAC* genes such as *JmNAC042*, *JmNAC055*, *JmNAC087*, *JmNAC090*, and *JmNAC091* contained exons and introns without the UTR region, indicating a specific evolutionary process of these genes.

**Figure 3:**
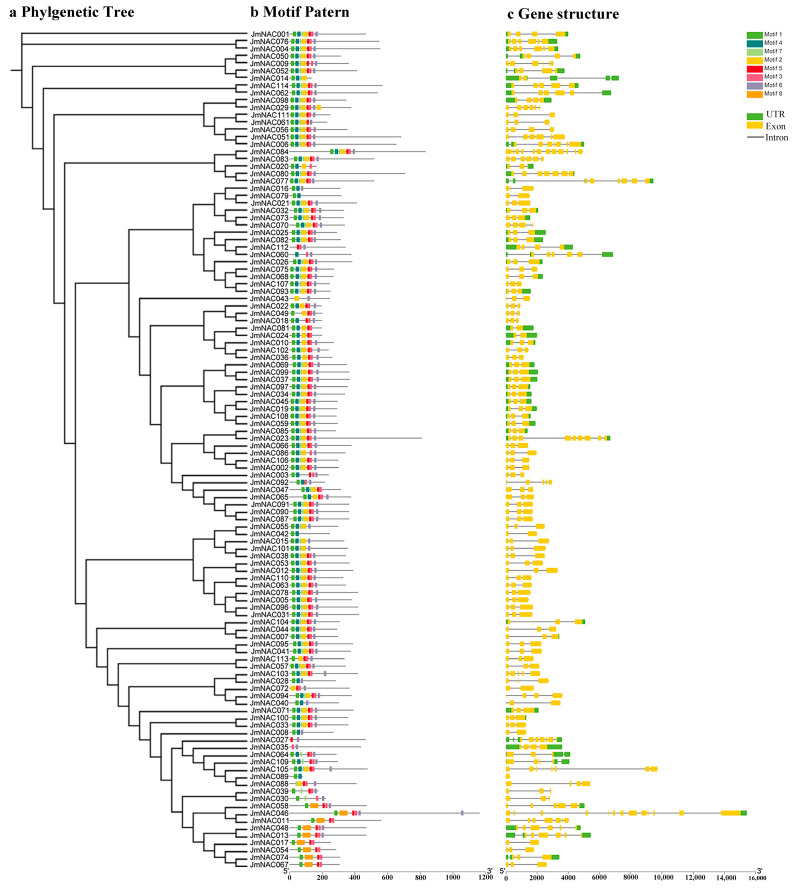
Phylogenetic relationships, gene structure and protein motifs in *JmNAC* genes. (**a**) The phylogenetic tree of all NAC proteins in *J. mandshurica* was constructed using the neighbour-joining method with 1000 replicates in the MEGA 7.0 software (Mega Limited, Auckland, New Zealand). (**b**) The motif composition of *J. mandshurica* NAC proteins. Motifs 1–10 are displayed in different coloured boxes. (**c**) CDS–UTR structure of *JmNAC* genes. Yellow boxes represent CDS; black lines indicate introns; green boxes represent the untranslated 5′ and 3′ regions.

Motifs are conserved sequences with biological functions, and different motifs usually have specific functional domains. To further analyze the functional regions of JmNAC proteins, the amino acid motifs of 114 JmNAC proteins were investigated using the MEME online tool (Figure 3). After MEME analysis, eight conserved motifs were predicted in *JmNAC* genes. A majority of *JmNAC* genes clustered into the same clade shared similar motif composition with the same position, indicating their similar biological functions. Motifs 1, 2, 4, 5, and 7 were found in most JmNAC proteins. Furthermore, most JmNAC proteins contained at least 5–6 conserved motifs, except for several specific proteins. A unique motif (motif 8) was identified in nine JmNAC proteins, which indicated that this specific motif was related to specific functions in different clades.

**Figure 4:**
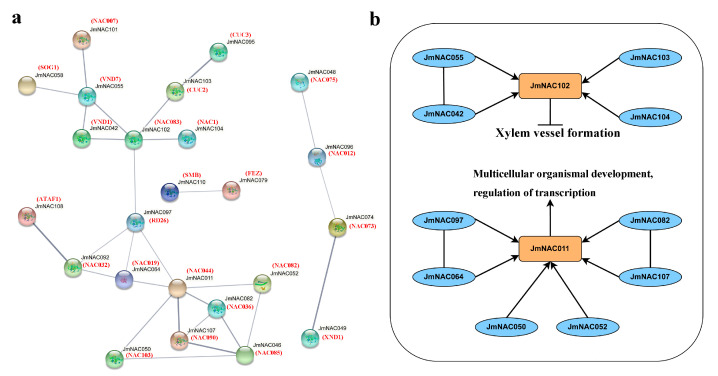
Functional network of *JmNAC* genes. (**a**) Network of *JmNAC* genes in *J. mandshurica* based on the orthologues in *A. thaliana*. (**b**) A schematic representation of a regulatory network among *JmNAC* genes.

To further analyze the potential function of *JmNAC* genes, an interaction network of 114 *JmNAC* genes was constructed using the STRING website tools according to the homologous proteins of *A. thaliana* (Figure 4). The results showed that five *JmNAC* genes including *JmNAC085*, *JmNAC011*, *JmNAC097*, *JmNAC102*, and *JmNAC055* may be the key nodes in this network, which interacted with other TFs to form a complicated regulatory network. A relatively high level of homology was found between *JmNAC102* and *AtNAC083*, which is a crucial transcriptional activator related to xylem vessel formation with a positive regulatory function. Furthermore, high homology was also found between *JmNAC011* and *AtNAC044*, which is a transcriptional regulator that positively regulates the expression of genes involved in the development of multicellular organisms. *JmNAC011* was closely related to *JmNAC097*, *JmNAC064*, *JmNAC050*, *JmNAC052*, *JmNAC107*, and *JmNAC082*, which may contribute to the formation of a strong interaction network.

### 2.4. Chromosomal Distribution and Synteny Analysis of JmNAC Genes

**Figure 5:**
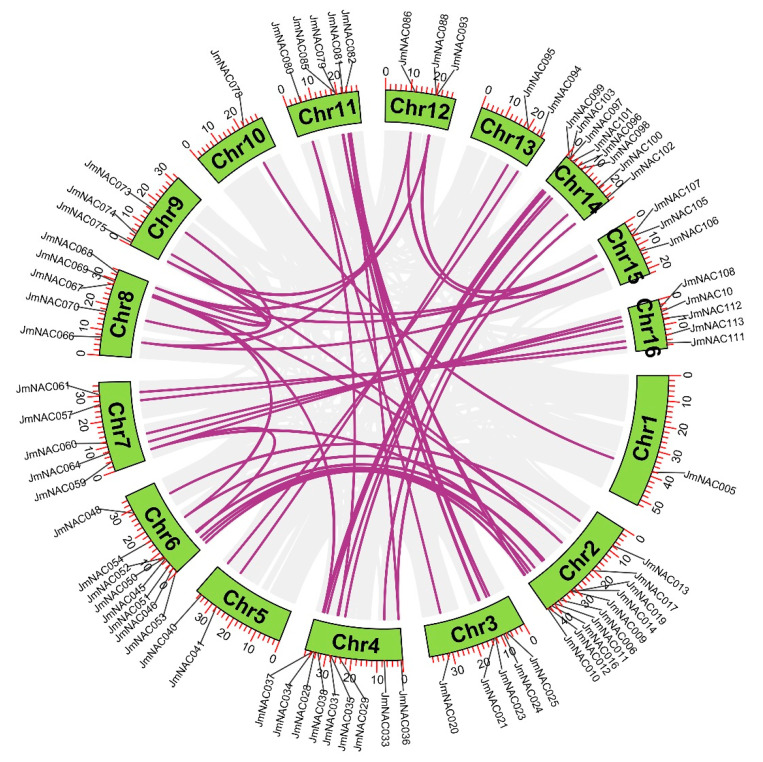
Genomic localization and gene duplication of *JmNAC* genes on *J. mandshurica* chromosomes. Grey lines indicate all syntenic blocks in the *J. mandshurica* genome; purple lines indicate the inter-chromosomal relationships of *JmNAC* genes.

Gene duplication events are common and widely occur in plant gene family formation, which is important for understanding the adaptive evolution of species. To understand the duplication events of all *JmNAC* genes, synteny analysis was performed using MCscanX and the Advanced Circos software in TBtools (Figure 5). The results revealed that a total of 50 orthologous gene pairs were identified in 114 *JmNAC* genes, indicating a close relationship between them. Furthermore, syntenic genes distributed on chromosome 2 (ten gene pairs) were the most common, followed by chromosome 4 (nine gene pairs) and chromosome 14 (eight gene pairs), whereas only one duplication gene pair was found on chromosomes 1 and 10. In addition, some *JmNAC* genes corresponded to more than one duplication gene pair on other chromosomes (Figure 5).

### 2.5. Syntenic Relationships of JmNAC Genes and NAC Genes of Several Different Species

To further analyze the evolutionary relationship between NAC genes of *J. mandshurica* and other different species including *Juglans regia*, *Juglans sigillata*, *Populus trichocarpa*, *A. thaliana* and *Vitis vinifera*, we performed comparative synteny analysis (Figure S1). *J. mandshurica*, *J. regia* and *J. sigillata* exhibited the highest level of homology. Furthermore, orthologous gene pairs in *J. mandshurica* and *J. regia* were mainly distributed on chromosomes 2 and 3, respectively. *J. mandshurica* and *J. sigillata* contained the largest number of orthologous gene pairs of chromosomes 6 and 3, respectively. *J. mandshurica* (orthologous gene pairs were mainly distributed on chromosome 2) and *P. trichocarpa* (orthologous gene pairs were mainly distributed on chromosome 1) also exhibited higher homology, whereas *J. mandshurica* (orthologous gene pairs were mainly distributed on chromosome 2) and *A. thaliana* (orthologous gene pairs were mainly distributed on chromosome 1) exhibited relatively low homology. Homology among NAC genes in *J. mandshurica* and other two genus *Juglans* plants (*J. regia* and *J. sigillata*) was higher than that in *J. mandshurica* and other several species, which may be related to species evolution and genetic relationships. Furthermore, we found that syntenic gene pairs on chromosome 2 were most abundant and diverse according to the intra- and inter-species collinearity analysis of 114 *JmNAC* genes. These results suggested that the NAC genes were relatively conserved in *J. mandshurica* and other plants.

### 2.6. Differential Expression of JmNAC Genes during Fruit Development in J. mandshurica

**Figure 6:**
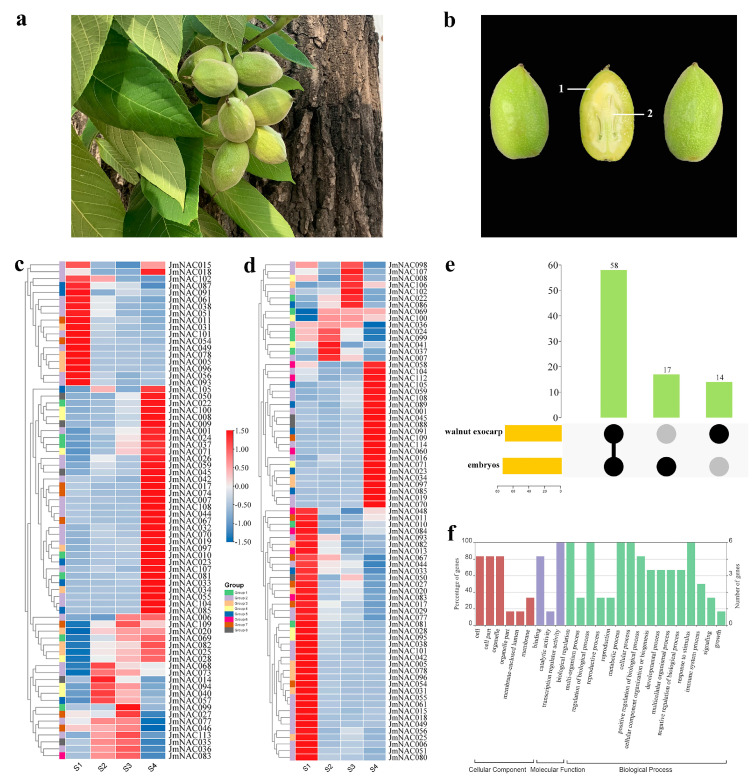
Expression profiles and functional analysis of *JmNAC* genes in different stages of fruit development and ripening. (**a**) Photographs of *J. mandshurica* fruits. (**b**) The tissue structure of *J. mandshurica* fruit. “1” indicates the walnut exocarp. “2” indicates the walnut embryos. (**c**) Heatmap representing 75 *JmNAC* gens in walnut exocarp at 30 days (S1), 50 days (S2), 70 days (S3) and 90 days (S4) after natural pollination. (**d**) Heatmap representing 75 *JmNAC* gens in walnut embryos at 30 days (S1), 50 days (S2), 70 days (S3) and 90 days (S4) after natural pollination. Group I–VIII correspond to the subgroups in the phylogenetic tree in Figure 2. (**e**) Upset plot of *JmNAC* genes expressed in walnut exocarp and embryos. (**f**) GO annotation analyses of the common *JmNAC* genes expressed in walnut exocarp and embryos.

Extensive studies have revealed that the NAC gene family plays a crucial role during fruit development and ripening stages. The expression pattern of all identified 114 *JmNAC* genes was further analyzed in walnut fruit (including exocarp and embryos) at different developmental stages (from S1 to S4 stages) combining the transcriptome data and RT-qPCR analysis (Figure 6). The expression levels of 114 *JmNAC* genes are shown in Figure 6. Our results revealed that most *JmNAC* genes exhibited different expression patterns in the two tissues (walnut exocarp and embryos) during fruit development. Among the identified genes, a total of 72 and 75 *JmNAC* genes were specifically expressed in walnut exocarp and embryos, respectively (Figure 6c,d), whereas 58 *JmNAC* genes were commonly expressed in the two tissues. A total of 17 and 14 *JmNAC* genes were specifically expressed in walnut exocarp and embryos, respectively (Figure 6e). To analyze the biological function of 58 genes expressed in all fruit samples, we performed function annotation analysis (Figure 6f). The GO analysis demonstrated that these genes were mainly divided into 25 functional categories, including 16 biological process (BP) categories, 6 cellular component (CC) categories and 3 molecular function (MF) categories. In the BP category, the most abundant terms were biological regulation (GO:0065007), regulation of biological process (GO:0050789) and metabolic process (GO:0008152). In the CC category, the most abundant term was cell and cell part. In the MF category, the most abundant term was transcription regulator activity (GO:0140110).

In walnut exocarp, a total of 39 *JmNAC* genes were significantly upregulated in the S4 stage, whereas 17 *JmNAC* genes exhibited higher expression levels in the S1 stage. A total of eight *JmNAC* genes (*JmNAC109*, *JmNAC020*, *JmNAC069*, *JmNAC082*, *JmNAC025*, *JmNAC028*, *JmNAC068*, and *JmNAC073*) exhibited higher expression levels in S2-S4 stages than that in the S1 stage during development, indicating their positive roles in fruit development. Among the identified subgroups, Group II contained the largest number of *JmNAC* genes that were upregulated or downregulated during the development of walnut exocarp. We found that 25 *JmNAC* genes exhibited high expression during the development of walnut embryos, especially in the S4 stage. In addition, a total of 35 *JmNAC* genes were significantly upregulated in the S1 stage. Furthermore, the expression of five *JmNAC* genes (*JmNAC024*, *JmNAC099*, *JmNAC041*, *JmNAC037*, and *JmNAC007*) and seven *JmNAC* genes (*JmNAC098*, *JmNAC107*, *JmNAC008*, *JmNAC106*, *JmNAC102*, *JmNAC022*, and *JmNAC086*) was relatively high in the S2 and S3 stages, respectively. Genes with high expression in the S4 stage were mainly derived from Group II. A total of 12 *JmNAC* genes with high expression were commonly expressed in the S4 stage in walnut exocarp and embryos.

### 2.7. Validation of Expression Pattern of RNA-seq Data Using RT-qPCR

**Figure 7:**
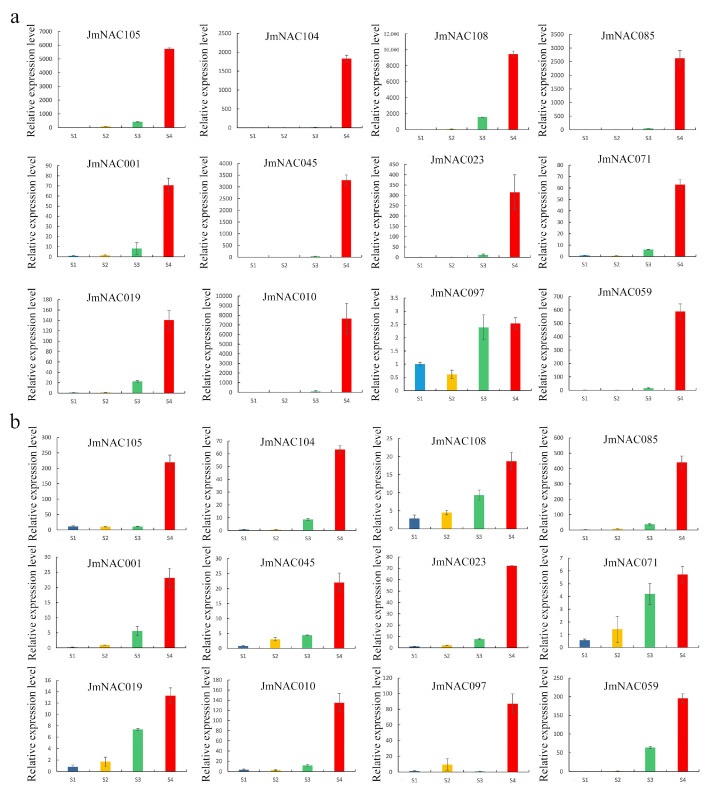
Expression profiles of 12 *JmNAC* genes during the development and ripening of fruits. (**a**) Relative expression of 12 *JmNAC* genes in walnut exocarp using RT-qPCR. (**b**) Relative expression of 12 *JmNAC* genes in walnut embryos using RT-qPCR. The *y*-axis shows the relative gene expression levels (2^−^^ΔΔ^^Ct^) analyzed by qRT-PCR. The *x*-axis represents the different tissue samples. The values are expressed as mean ± standard deviation of three replicates.

To better understand the expression pattern of *JmNAC* genes during the development and ripening of fruits, 12 potential *JmNAC* genes from four developmental stages of walnut exocarp and embryos were selected and used for RT-qPCR analysis with specific primers (Table S2). As demonstrated in Figure 7, all selected *JmNAC* genes exhibited relatively high expression in the S4 stage in the two different tissues, which is consistent with the results obtained from RNA-seq analysis. Furthermore, the expression level of six *JmNAC* genes, including *JmNAC105*, *JmNAC104*, *JmNAC085*, *JmNAC045*, *JmNAC010*, and *JmNAC023*, in the S4 stage was significantly higher than that in the S1 stage, which indicated a positive role of *JmNAC* genes during the development of fruits. Therefore, RT-qPCR results supported the credibility and accuracy of RNA-seq data in this study.

## 3. Discussion

*J. mandshurica* is a well-known and valuable tree species for its elite hardwood, edible seed kernel and extensive medicinal value of walnut exocarp [46,47]. However, only a few studies have been conducted on its growth, development, and stress response at molecular level due to the lack of genome sequence and RNA-seq data. Previous studies have found that TFs are powerful tools for regulating plant development and enhancing plant tolerance to environmental stresses such as drought, low temperature, high salinity, and high temperature. NAC TFs are one of the largest plant-specific TF families in plants and play a vital role in regulating plant senescence, metabolite synthesis, fruit development and signal transduction. Genome-wide identification and analysis are indispensable methods for investigating specific functions of the NAC gene family. To date, some NAC family members have been identified and characterized in plant species such as *A. thaliana* [21,48], *Pyrus bretschneideri* [49], *Jatropha curcas* [50], and *Sorghum bicolor* [51]. However, genome-wide analysis of the gene family of *J. mandshurica* has not been performed, and the regulatory function of *JmNAC* genes remains unclear. In the present study, the identification and expression analysis of the NAC family at the genome level provided genetic information and a better understanding of the development of walnut exocarp and embryo.

The number and structural features of TFs in a gene family are related not only to the genome size of the species but also to the influence of long-term evolution of plants. In this study, we identified a total of 114 genes encoding NAC protein in the *J. mandshurica* genome (Table S1). The number of NAC genes was significantly higher in *J. mandshurica* than that in *Panax ginseng* (89 *PgNACs* genes) [43], *Phyllostachys heterocycla* (94 *PeNACs* genes) [52], *Xanthoceras sorbifolia* (103 *XsNACs* genes) [26] and *J. regia* (102 *JrNACs* genes) [53] but was the same as in *Betula pendula* (114 *BpNACs* genes) [54] and *Dimocarpus longan* (114 *DlNACs* genes) [44], indicating high conservation of NAC gene family in *J. mandshurica*, which may be related to gene duplication during species formation and evolution. With regard to subcellular localization, all 114 *JmNACs* were predicted and located in the nucleus, which may be closely related to the expression regulation of target genes. Furthermore, the exons and introns of identified *JmNAC* genes varied from 3–13 and 1–2, respectively, wherein genes on the same branch displayed similar organization of exons and introns (Figure 3). Most *JmNAC* genes consist of three exons, similar to *J. regia* [53], *Cucumis sativus* [55] and *Chenopodium quinoa* [56], which may be related to the functional differentiation and structural diversity of the *JmNAC* gene family. Although there were some differences in the arrangement of conserved motifs, numerous *JmNAC* genes shared similar motif composition, indicating similar structure and biological function. These results confirm the characteristics of the *JmNAC* gene family and facilitate further study on the function of *JmNAC* genes.

Investigating the origin and differentiation of gene families is a prime research focus of evolutionary studies. Identifying a gene family based on the whole genome data is of great significance for understanding gene evolution history, gene function and species differentiation [57,58]. According to the phylogenetic tree analysis of NAC genes in *J. mandshurica* and *A. thaliana*, 114 *JmNACs* were significantly clustered into eight subgroups (Figure 2), wherein Group I contained the largest number of *JmNAC* and *AtNAC* genes, and the genes from Group I mainly involved in DNA-binding TF activity, embryo development, leaf senescence, signal transduction, oxidative stress response, and xylem formation, indicating various functions of *JmNAC* genes in plant growth and development as well as stress response. Also, the findings showed that the NAC members from the same subfamily share a similar functional characteristic, however, further functional feature need to be confirmed. Differences in the NAC family members between *J. mandshurica* and *A. thaliana* in different subgroups may be owing to gene differentiation caused by the continuously changing environment during evolution.

Previous studies have confirmed that gene duplication plays a crucial role in adaptive evolution of plants. Many plants have experienced evolutionary events such as large fragments and tandem and whole genome duplication, which contribute to the formation and rapid expansion of gene families. Furthermore, to adapt to the drastically changing external environment and avoid destructive extinction, high-level gene duplication events may occur in many gene families such as WRKY, MYB, and MADS-box TFs. In the present study, four tandemly duplicated gene pairs were identified, suggesting that the expansion of the NAC gene family might have been originated from genome polyploidy events that contributed to diversify gene function. In addition, among the identified 114 *JmNACs*, we obtained 50 orthologous gene pairs using synteny analysis (Figure 5), indicating that *J. mandshurica* may undergo multiple selecting evolutionary directions. *JmNAC93*, *JmNAC106* and *JmNAC107* had more than one orthologous gene pair, which further supports the fact that they play a crucial role in the adaptation of plants to the changing terrestrial environment.

Gene family members have formatted protein complexes and diverse PPIs during long evolutionary processes. Analysis of the PPT network indicated that *JmNACs* were widely involved in tissue formation (Figure 4). A total of five key hub genes including *JmNAC055*, *JmNAC085*, *JmNAC097*, *JmNAC011*, and *JmNAC102* were found in the interaction network, and they could be used as candidate gene for studying tissue development and formation of *J. mandshurica*. *JmNAC085* and *JmNAC097* were significantly upregulated in the S4 stage, indicating that they may be positive regulators and exhibited tissue-specific function for regulating the development and maturation of walnut exocarp and embryo and could be used as candidate genes in future study on fruit development of *J. mandshurica*. These results further enriched the molecular mechanism of NAC transcription factor for regulating *J. mandshurica* fruit development.

Fruit development is a complex biological process that involves nutrient formation and secondary product metabolism, directly affecting fruit quality formation and commodity value [59]. Previous studies have found that the development, maturation, senescence and quality formation of fruits are specifically regulated by many environmental and regulatory factors, especially NAC TFs [38]. A lot of attention has been paid to the role of the NAC gene family members during fruit development and ripening. A recent study found that *PpNAC1* regulated the expression of ripening-related *PpAAT1* and contributed to volatile ester formation in *Prunus persica* [60], indicating a key role during fruit ripening. In papaya (*Carica papaya*), *CpNAC1* was reported to regulate carotenoid accumulation during fruit ripening [61]. Similar results were reported in studies on *Actinidia eriantha* [45], *Pyrus pyrifolia* [62], *Malus domestica* [63], *Eriobotrya japonica* [64], and other fruit tree species. In addition, NAC TFs also regulate fruit development and ripening through multiple hormone such as ethylene and abscisic acid biosynthesis and signal transduction pathways [65]. For instances, in tomato, *SNAC4* and *SNAC9* could positively regulate the fruit ripening process by interacting with ethylene synthesis genes [66]. Also, the expression patterns of the *SNAC* genes were affected by abscisic acid in tomato [67]. However, the regulatory function of NAC TFs during fruit development in *J. mandshurica* remains unknown. The expression profile analysis of NAC gene in different tissues and developmental stages of *J. mandshurica* provides new data for understanding the potential biological functions of NAC gene in *J. mandshurica*. In the present study, the process of *J. mandshurica* fruit ripening is accompanied by the gene expression and TFs regulation. Expression analyses of all identified 114 *JmNACs* during different developmental stages in *J. mandshurica* revealed that most genes may have possible functions during fruit development and ripening. Particularly, according to the expression analysis, we found that most of the same subfamily genes displayed the similar expression patterns in two tissues, but small amounts of genes from the same subfamilies also showed differential expression level, which may be caused by the functional differentiation of *JmNACs*. A total of 72 (63.16%) and 75 (65.79%) *JmNAC* genes were specifically expressed during fruit development in walnut exocarp and embryos (Figure 6). Furthermore, 39 and 25 key *JmNAC* genes were significantly upregulated in the S4 stage in walnut exocarp and embryos, respectively, indicating that they specifically regulated the development of walnut exocarp and embryos. We also identified 12 key hub *JmNAC* genes that exhibited constitutive high expression levels during ripening stages in walnut exocarp and embryos, and RT-qPCR analysis also confirmed the results (Figure 7), inferring a strict regulation of fruit development. Therefore, we predict that these key *JmNAC* genes above will offer new avenues for exploring the role of NAC TFs in fruit growth and development, and their genetic functions require further characterization. Further validation of their functions will greatly advance our understanding of fruit development in *J. mandshurica*.

## 4. Materials and Methods

### 4.1. Plant Materials

The fruit of *J. mandshurica* used in this study was collected from the Northeast Forestry University (126°37′57.28″ E, 45°43′6.53″ N), Harbin, Heilongjiang province, China. From June to September, the fruits with strong growth and no pests or diseases were sampled and used for RNA extraction. Walnut exocarp and embryos were collected from four stages, at 30 days (S1 stage), 50 days (S2 stage), 70 days (S3 stage), and 90 days (S4 stage) after natural pollination, and were used for real-time reverse transcription polymerase chain reaction (RT-qPCR). In which, S1, S2, and S3 indicate the different developmental stages, while the S4 represents the ripening stages of Walnut fruits. For each stage, the sampling time (2:00 p.m.) of collected fruits was consistent. All sampled fruits were rapidly frozen in liquid nitrogen and subsequently stored at −80 °C for RNA extraction.

### 4.2. Identification of JmNAC Genes in J. mandshurica Genome

To perform genome-wide identification of the *JmNAC* gene family, the whole genome sequence and annotation data were accessed from our previous genomic study, and the accession number of the Genome Warehouse in National Genomics Data Center (NGDC) (https://ngdc.cncb.ac.cn/) (accessed on 6 October 2021) was PRJCA006358. We assembled a high-quality chromosome-scale reference genome assembly and annotation for *J. mandshurica* (n = 16) with a contig N50 of 21 Mb and BUSCO complete gene percentage of 98.3% by combining PacBio high-fidelity (HiFi) reads with high-throughput chromosome conformation capture (Hi-C) data. The genome obtained is of sufficient quality for genome-wide studies and expression analysis of NAC gene families and gene function in *J. mandshurica*. The *A. thaliana* NAC full-length protein sequences were obtained from The Arabidopsis Information Resource database (TAIR; https://www.arabidopsis.org/) (accessed on 4 August 2021). First, *JmNAC* genes in *J. mandshurica* were identified using two BLAST methods. A total of 96 *AtNAC* genes were used as reference sequences to search the possible *JmNAC* genes using BLAST (e-value, 1 × e^−5^) in the TBtools software (version 1.087) [68]. Subsequently, all genes retrieved using BLAST were further identified using Batch NCBI CD-Search Tools (https://www.ncbi.nlm.nih.gov/Structure/bwrpsb/bwrpsb.cgi) (accessed on 4 August 2021) to obtain the conserved NAC domains. Lastly, the Pfam (http://pfam.xfam.org/) (accessed on 6 August 2021) and SMART (http://smart.embl-heidelberg.de/) (accessed on 6 August 2021) databases were used to verify the final candidate *JmNAC* genes.

### 4.3. Sequence Analysis and Gene Structural Characterisation

The number of amino acids and length of coding sequences (CDS) were calculated using the TBtools software [68]. Molecular weight (*M*_W_) and isoelectric point (pI) of each NAC protein were analysed using the online ExPASy program (https://web.expasy.org/protparam/) (accessed on 8 August 2021). Subcellular localisation of all JmNAC proteins was performed using the Cell-PLoc 2.0 web tool (http://www.friendbio.com/meansMore/id/52) (accessed on 8 August 2021). To investigate the gene motifs of JmNAC proteins, MEME (http://meme-suite.org/tools/meme) (accessed on 10 August 2021) was used, with the parameters set at maximum eight motifs. To further visualise the motif composition and gene structure, integration analysis was conducted using TBtools [68].

### 4.4. Chromosomal Location and Evolutionary Analysis of JmNAC Genes

For identifying chromosomal locations, the TBtools software was used to locate and visually map *JmNAC* genes on 16 chromosomes in *J. mandshurica* based on the General Feature Format (GFF) information. Furthermore, tandemly duplicated gene pairs were marked with a red line. The names of candidate *JmNAC* genes were encoded according to the physical location information of chromosomes. To further analyse gene replication, duplicated and orthologous pairs of *JmNAC* genes were obtained by constructing a dual synteny plot in MCscanX, and the Advanced Circos software (https://github.com/CJ-Chen/TBtools) (accessed on 15 August 2021) was used to display the collinearity circle plot. Homology among NAC genes in *J. mandshurica* and five representative plant species including *J. regia*, *J. sigillata*, *P. trichocarpa*, *A. thaliana* and *V. vinifera* was inferred using the multiple collinearity scan toolkit (MCScanX, http://chibba.pgml.uga.edu/mcscan2/) (accessed on 15 August 2021) with default parameters of the TBtools software.

### 4.5. Chromosomal Location and Evolutionary Analysis of JmNAC Genes

To further demonstrate the evolutionary relationship between NAC proteins of *J. mandshurica* and *A. thaliana*, multiple sequence alignment was performed using the ClustalW algorithm in MEGA (version 7.0) software [69] with the following parameters: pairwise deletion and 1000 replicates for bootstrap analysis. Subsequently, the alignment results were used as input files to construct a phylogenetic tree using neighbour-joining methods. For graphic display, the final phylogenetic tree of the JmNAC gene family was further processed using the Interactive Tree Of Life (iTOL) (https://itol.embl.de/) (accessed on 20 August 2021) web tools. NAC proteins from *J. mandshurica* and *A. thaliana* were eventually divided into different subgroups.

### 4.6. Interaction Network Construction of JmNAC Genes

To predict the function of JmNAC proteins, protein–protein interaction (PPI) networks were constructed on the STRING website (https://www.string-db.org/) (accessed on 20 August 2021). Briefly, the amino acid sequences of JmNAC proteins were used to scan the representative AtNAC proteins using BLASTP in TBtools. Subsequently, the candidate sequences were uploaded on the STRING website to construct an interaction network of *JmNAC* genes with default parameters.

### 4.7. Expression Analysis of JmNAC Genes during the Development and Ripening of Fruits

To analyze the expression patterns of *JmNAC* genes in different developmental stages of fruits of *J. mandshurica*, the raw RNA-seq reads of two tissues including walnut exocarp and embryos were obtained from the National Center for Biotechnology Information (NCBI; http://www.ncbi.nlm.nih.gov/) (accessed on 9 October 2021) sequence read archive (SRA) database (PRJNA733587). After quality control, alignment and quantitative analysis, the count matrix was obtained and used to calculate the expression of *JmNAC* genes in reads per kilobase per million (RPKM). Subsequently, the normalized data were further used to obtain the expression heatmap of all *JmNAC* genes involved in the development and ripening of fruits in *J. mandshurica* using the Heatmap program in TBtools. Furthermore, the common *JmNAC* genes in walnut exocarp and embryos were selected and annotated to the Gene Ontology (GO) database using the eggnog database (http://eggnog-mapper.embl.de/) (accessed on 5 October 2021).

### 4.8. RNA Extraction and RT-qPCR

The total RNA in four developmental stages of walnut exocarp and embryos was extracted using the plant total RNA extraction kit (Takara, Beijing, China) according to the manufacturer’s instructions. Subsequently, the integrity and quality of total RNA of all tissue samples were assessed using agarose gel electrophoresis and the K5500 plus microspectrophotometer (KAIAO Technology Development Co., Ltd., Beijing, China), respectively. Approximately 1 μg of total RNA was used for synthesising cDNA using the PrimeScript RT reagent kit with gDNA Eraser (TaKaRa, Kyoto, Japan), and the amplification products were used for qRT-PCR analysis. The qRT-PCR primers were designed using Primer3web tools (version 4.1.0; https://primer3.ut.ee/) (accessed on 5 September 2021). Furthermore, 18s RNA was used as an internal reference gene. A total of 12 key DEGs were selected and used for RT-qPCR analysis with specific primers (Table S2). Real-time PCR was performed in triplicates on the ABI 7500 Fast Real-Time Detection System using the TaKaRa SYBR Green Mix kit (TaKaRa, Beijing, China). The PCR reaction was performed in a solution of 20 µL, which contained 10 µL of 2× SYBR Premix Ex Taq, 6 µL of double-distilled water (ddH_2_O), 2 µL of cDNA template, 0.8 µL of upstream and downstream primers (10 μmol/L) and 0.4 µL of ROX reference dye. The PCR reaction conditions were set as follows: 95 °C for 30 s, 40 cycles at 95 °C for 5 s, 60 °C for 35 s and 95 °C for 15 s, 60 °C for 1 min, followed by 95 °C for 15 s. The relative expression level was assessed using the 2^−∆∆CT^ method. A histogram was created based on the expression data of qRT-PCR.

## Data Availability

The transcriptome raw datasets (two tissues for four stages with triplicates) were deposited in the National Center for Biotechnology Information (NCBI) Sequences Read Archive (SRA) (https://www.ncbi.nlm.nih.gov/bioproject/?term=prjna733587) (accessed on 29 May 2021) under the accession number PRJNA733587 (including embryos and exocarp).

## Supplementary Materials

The following are available online at https://www.mdpi.com/article/10.3390/ijms222212414/s1.

## Abbreviations

NAC	NAM, ATAF and CUC
TFs	transcription factors
CDS	coding sequence
*M*_W_	molecular weight
pI	isoelectric point
GO	Gene Ontology
RPKM	reads per kilobase per million
qRT-PCR	Quantitative real-time polymerase chain reaction
