# Supplementary material for: Genome-Wide Identification of NAC Transcription Factor Family in Juglans mandshurica and Their Expression Analysis during the Fruit Development and Ripening
Source: Int J Mol Sci. 2021 Nov 17;22(22):12414. doi: 10.3390/ijms222212414 (PMC8625062; doi:10.3390/ijms222212414)
Supplement: Supplementary file 1 [file ijms-22-12414-s001.zip › ijms-1438165-supplementary.pdf]

International Journal of
Molecular Sciences

MDPI

Table S1. Basic information of the *NAC* genes in *Juglans mandshurica*.

Gene ID	Gene name	Number of amino acids	Molecular weight (MW)	Theoretical pI(pl)	CDS Length	SL	Chromosome
rna-Jman001T0042200.1	JmNAC001	464	51770.78	5.92	1395	Nucleus	1
rna-Jman001T0254600.1	JmNAC002	294	33902.24	8.36	894	Nucleus	1
rna-Jman001T0145400.1	JmNAC003	236	27427.79	5.46	711	Nucleus	1
rna-Jman001T0042300.1	JmNAC004	552	62027.19	4.58	1659	Nucleus	1
rna-Jman001T0289300.1	JmNAC005	378	43706.81	5.74	1137	Nucleus	1
rna-Jman002T0279700.1	JmNAC006	651	74177.15	5.56	1956	Nucleus	2
rna-Jman002T0020400.1	JmNAC007	294	33858.11	5.87	885	Nucleus	2
rna-Jman002T0331500.1	JmNAC008	265	29434.45	4.85	798	Nucleus	2
rna-Jman002T0228100.1	JmNAC009	359	40114.85	4.83	1080	Nucleus	2
rna-Jman002T0349500.1	JmNAC010	267	30729.09	9.31	804	Nucleus	2
rna-Jman002T0309700.1	JmNAC011	558	61752.87	6.62	1677	Nucleus	2
rna-Jman002T0344800.1	JmNAC012	387	43760.68	6.74	1164	Nucleus	2
rna-Jman002T0069800.1	JmNAC013	466	52477.28	6.6	1401	Nucleus	2
rna-Jman002T0226100.1	JmNAC014	132	15770.41	9.6	399	Nucleus	2
rna-Jman002T0260300.1	JmNAC015	332	38409.99	6.31	999	Nucleus	2
rna-Jman002T0328200.1	JmNAC016	309	34511.73	4.88	930	Nucleus	2
rna-Jman002T0139300.1	JmNAC017	249	28051.61	9.41	750	Nucleus	2
rna-Jman002T0231700.1	JmNAC018	195	22452.25	4.81	588	Nucleus	2

rna-Jman002T0235800.1	JmNAC019	289	33329.81	6.02	870	Nucleus	2
rna-Jman003T0241400.1	JmNAC020	162	19075.76	6.31	489	Nucleus	3
rna-Jman003T0136500.1	JmNAC021	408	45971.03	7.13	1227	Nucleus	3
rna-Jman003T0037700.1	JmNAC022	192	22074.6	5.35	579	Nucleus	3
rna-Jman003T0132000.1	JmNAC023	807	92614.04	6.74	2424	Nucleus	3
rna-Jman003T0100700.1	JmNAC024	196	22570.87	9.65	591	Nucleus	3
rna-Jman003T0086200.1	JmNAC025	287	33260.96	7.78	864	Nucleus	3
rna-Jman003T0000600.1	JmNAC026	381	43553.53	6.77	1146	Nucleus	3
rna-Jman003T0242400.1	JmNAC027	462	50582.53	4.46	1389	Nucleus	3
rna-Jman004T0223600.1	JmNAC028	281	30065.44	4.92	846	Nucleus	4
rna-Jman004T0139000.1	JmNAC029	374	42724.88	5.09	1125	Nucleus	4
rna-Jman004T0062200.1	JmNAC030	218	25545.17	8.59	657	Nucleus	4
rna-Jman004T0164800.1	JmNAC031	422	48001.29	5.81	1269	Nucleus	4
rna-Jman004T0088300.1	JmNAC032	330	37780.19	8.44	993	Nucleus	4
rna-Jman004T0056500.1	JmNAC033	357	40268.52	7.03	1074	Nucleus	4
rna-Jman004T0231600.1	JmNAC034	336	38454.06	7.7	1011	Nucleus	4
rna-Jman004T0161100.1	JmNAC035	436	48451.99	4.75	1311	Nucleus	4
rna-Jman004T0004400.1	JmNAC036	260	29123.95	7.58	783	Nucleus	4
rna-Jman004T0231700.1	JmNAC037	365	40462.66	8.15	1098	Nucleus	4
rna-Jman004T0208200.1	JmNAC038	343	40011.77	6.24	1032	Nucleus	4

rna-Jman004T0062100.1	JmNAC039	172	20117.74	8.88	519	Nucleus	4
rna-Jman005T0267700.1	JmNAC040	299	32542.32	6.14	900	Nucleus	5
rna-Jman005T0188200.1	JmNAC041	373	41718.13	5.6	1122	Nucleus	5
rna-Jman005T0066100.1	JmNAC042	244	28028.3	4.57	735	Nucleus	5
rna-Jman005T0151200.1	JmNAC043	243	27671.1	5.28	732	Nucleus	5
rna-Jman006T0241600.1	JmNAC044	289	33285.52	5.66	870	Nucleus	6
rna-Jman006T0081100.1	JmNAC045	290	33199.74	7.01	873	Nucleus	6
rna-Jman006T0029000.1	JmNAC046	1160	132712.83	5.36	3483	Nucleus	6
rna-Jman006T0194000.1	JmNAC047	311	35149.96	5.75	936	Nucleus	6
rna-Jman006T0195700.1	JmNAC048	467	52688.32	6.43	1404	Nucleus	6
rna-Jman006T0084400.1	JmNAC049	198	22937.79	4.82	597	Nucleus	6
rna-Jman006T0085500.1	JmNAC050	310	35866.12	6.28	933	Nucleus	6
rna-Jman006T0049900.1	JmNAC051	681	77796.91	5.41	2046	Nucleus	6
rna-Jman006T0086300.1	JmNAC052	411	45629.43	4.61	1236	Nucleus	6
rna-Jman006T0007200.1	JmNAC053	365	42030.85	7	1098	Nucleus	6
rna-Jman006T0145600.1	JmNAC054	282	31947.14	9.04	849	Nucleus	6
rna-Jman007T0092600.1	JmNAC055	295	33968.83	5.99	888	Nucleus	7
rna-Jman007T0132600.1	JmNAC056	351	40185.7	4.97	1056	Nucleus	7
rna-Jman007T0160300.1	JmNAC057	341	38328.96	8.29	1026	Nucleus	7
rna-Jman007T0004500.1	JmNAC058	468	52902.03	5.01	1407	Nucleus	7

rna-Jman007T0032300.1	JmNAC059	291	33328.8	6.47	876	Nucleus	7
rna-Jman007T0081000.1	JmNAC060	375	41669.51	7.04	1128	Nucleus	7
rna-Jman007T0183200.1	JmNAC061	228	25996.11	5.06	687	Nucleus	7
rna-Jman007T0010900.1	JmNAC062	538	61196.44	4.8	1617	Nucleus	7
rna-Jman007T0128700.1	JmNAC063	343	39394.17	7.23	1032	Nucleus	7
rna-Jman007T0058200.1	JmNAC064	285	31801.86	6.15	858	Nucleus	7
rna-Jman008T0090700.1	JmNAC065	374	41947.89	9.18	1125	Nucleus	8
rna-Jman008T0060700.1	JmNAC066	376	43031.34	6.5	1131	Nucleus	8
rna-Jman008T0205900.1	JmNAC067	303	34277.44	6.54	912	Nucleus	8
rna-Jman008T0256800.1	JmNAC068	265	30268.81	6.01	798	Nucleus	8
rna-Jman008T0216000.1	JmNAC069	347	38155.58	8.42	1044	Nucleus	8
rna-Jman008T0111400.1	JmNAC070	335	38413.39	8.72	1008	Nucleus	8
rna-Jman009T0000400.1	JmNAC071	388	43665.14	6.36	1167	Nucleus	9
rna-Jman009T0034100.1	JmNAC072	364	41481.46	5.34	1095	Nucleus	9
rna-Jman009T0135400.1	JmNAC073	327	37286.09	8.29	984	Nucleus	9
rna-Jman009T0055100.1	JmNAC074	307	34822.89	6.95	924	Nucleus	9
rna-Jman009T0012900.1	JmNAC075	269	30686.87	5.56	810	Nucleus	9
rna-Jman010T0030200.1	JmNAC076	547	61674.8	4.65	1644	Nucleus	10
rna-Jman010T0173400.1	JmNAC077	517	59629.45	6.28	1554	Nucleus	10
rna-Jman010T0176100.1	JmNAC078	416	46879.98	5.87	1251	Nucleus	10

rna-Jman011T0121400.1	JmNAC079	314	35194.61	5.16	945	Nu cle us	11
rna-Jman011T0042100.1	JmNAC080	706	77661.55	5.06	2121	Nu cle us	11
rna-Jman011T0147600.1	JmNAC081	194	22380.84	9.83	585	Nu cle us	11
rna-Jman011T0160100.1	JmNAC082	311	35891.96	6.11	936	Nu cle us	11
rna-Jman011T0042200.1	JmNAC083	516	57091.17	4.52	1551	Nu cle us	11
rna-Jman011T0042400.1	JmNAC084	831	93936.83	4.92	2496	Nu cle us	11
rna-Jman011T0124700.1	JmNAC085	280	32251.37	8.22	843	Nu cle us	11
rna-Jman012T0120300.1	JmNAC086	339	38719.67	8.27	1020	Nu cle us	12
rna-Jman012T0193900.1	JmNAC087	362	40968.35	4.74	1089	Nu cle us	12
rna-Jman012T0162800.1	JmNAC088	407	45992.82	6.62	1224	Nu cle us	12
rna-Jman012T0162900.1	JmNAC089	77	8773.86	4.21	234	Nu cle us	12
rna-Jman012T0194400.1	JmNAC090	362	40937.34	4.74	1089	Nu cle us	12
rna-Jman012T0194200.1	JmNAC091	362	41013.35	4.74	1089	Nu cle us	12
rna-Jman012T0185900.1	JmNAC092	213	24127.68	7.66	642	Nu cle us	12
rna-Jman012T0164200.1	JmNAC093	362	41013.35	4.74	747	Nu cle us	12
rna-Jman013T0187000.1	JmNAC094	378	41860.33	9.31	1137	Nu cle us	13
rna-Jman013T0129100.1	JmNAC095	386	43392.15	6.8	1161	Nu cle us	13
rna-Jman014T0070800.1	JmNAC096	416	47477.7	5.62	1251	Nu cle us	14
rna-Jman014T0022400.1	JmNAC097	353	40089.96	6.76	1062	Nu cle us	14
rna-Jman014T0086200.1	JmNAC098	344	38987.75	5.47	1035	Nu cle us	14

rna-Jman014T0022300.1	JmNAC099	362	40123.14	8.84	1089	Nucleus	14
rna-Jman014T0154400.1	JmNAC100	356	40306.47	8.25	1071	Nucleus	14
rna-Jman014T0038300.1	JmNAC101	355	41498.14	6.48	1068	Nucleus	14
rna-Jman014T0175400.1	JmNAC102	236	26646.9	8.62	711	Nucleus	14
rna-Jman014T0027000.1	JmNAC103	415	45933.85	8.93	1248	Nucleus	14
rna-Jman014T0158600.1	JmNAC104	304	34249.64	6.71	915	Nucleus	14
rna-Jman015T0044000.1	JmNAC105	475	52812.87	8.56	1428	Nucleus	15
rna-Jman015T0072700.1	JmNAC106	297	34079.45	8.36	894	Nucleus	15
rna-Jman015T0042500.1	JmNAC107	243	27616.14	6.46	732	Nucleus	15
rna-Jman016T0028600.1	JmNAC108	285	32441.76	8.42	858	Nucleus	16
rna-Jman016T0046500.1	JmNAC109	292	32490.67	6.12	879	Nucleus	16
rna-Jman016T0085400.1	JmNAC110	325	37219.97	8.09	978	Nucleus	16
rna-Jman016T0115200.1	JmNAC111	246	28556.07	5.26	741	Nucleus	16
rna-Jman016T0062100.1	JmNAC112	340	37481.86	4.87	1023	Nucleus	16
rna-Jman016T0096400.1	JmNAC113	333	37809.36	6.45	1002	Nucleus	16
rna-Jman016T0006600.1	JmNAC114	566	63625.84	5.02	1701	Nucleus	16

Table S2. Primer sequences of *J. mandshurica.*

Gene name	Forward primer (F)	Reverse primer (R)
18s RNA	GAGGTAGCTTCGGGCGCAACT	GCAGGTTAGCGAAATGCGATAC
JmNAC105	GACCCATCAGCTTTCCTCTTCACCC	AGTCAGGCCATCTCTCAACCTCAGT
JmNAC104	ATGCGATTACTTGATGGGGAAGGCC	ACCAAGATGATGATGAGGGGATCGA
JmNAC108	CGGTGGCGATGGTCCCTTTCTTAAA	TTGCTTCCCGATACTGCCCTTCTTG
JmNAC085	TGGTACTTCTTTAGCCCGCGTGATC	AAACACCAGAGCTTTCTTCACCCCA
JmNAC001	CGGTTTCTCCTCCATTTGATCCTGCT	GCGAATCATGGTTTGGGCACTCATC
JmNAC045	TGCGTCGTGGGTCAAGCTTAATCTT	CCACTCCTTTTCACCGTACAGAGCC
JmNAC023	AACGAGTGGTACTTCTTTAGCCCGC	ACACAAGAGCTTTCTTCACCCCAACA
JmNAC071	TGGGTTGTCTGCAGAGTTTTCCACA	ACAGCTAGGCCTTTCGTTGTTGGAA
JmNAC019	TCATCAATGATCACCGGCGACCAAT	TCTCCTCCCACTTGGCTTCACTTTG
JmNAC010	CTCTATCCCATTGCCGGCTTCAGTC	ACAAACACACACACACACACACCT
JmNAC097	TCAAAGTGAACCCATAACTCAGCGG	TTTTACTGTCGGAACCTCGGAACCC
JmNAC059	TCTTTTCGCCTAGGGACCGGAAGTA	TGCCGGAGTAAAACACAAGAGCCTT

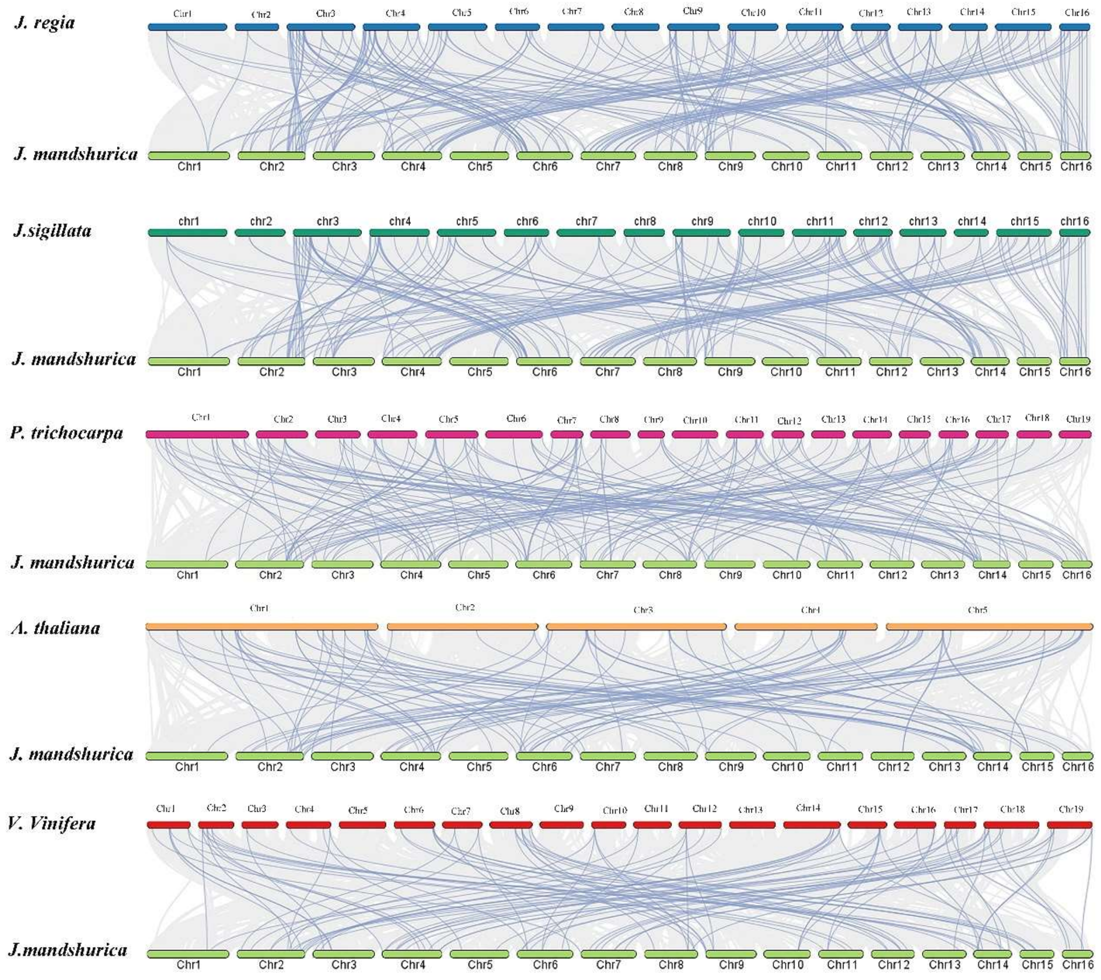

Figure S1. Synteny analysis of NAC genes in *J. mandshurica* and five representative plant species. Grey lines in the background indicate collinear blocks within *J. mandshurica* and other plant genomes, whereas blue lines highlight syntenic *JmNAC* gene pairs.
